# Correction: Effects of short-term resistance training and tapering on maximal strength, peak power, throwing ball velocity, and sprint performance in handball players

**DOI:** 10.1371/journal.pone.0221189

**Published:** 2019-08-09

**Authors:** Souhail Hermassi, Aloui Ghaith, René Schwesig, Roy J. Shephard, Mohamed Souhaiel Chelly

The following information is missing from the Funding statement: The publication of this article was funded by the Qatar National Library. No additional external funding was received for this study.

The histograms are partially hidden in Figs 3, 4 and 5. Please see the correct Figs 3, 4 and 5 here.

**Fig 3:**
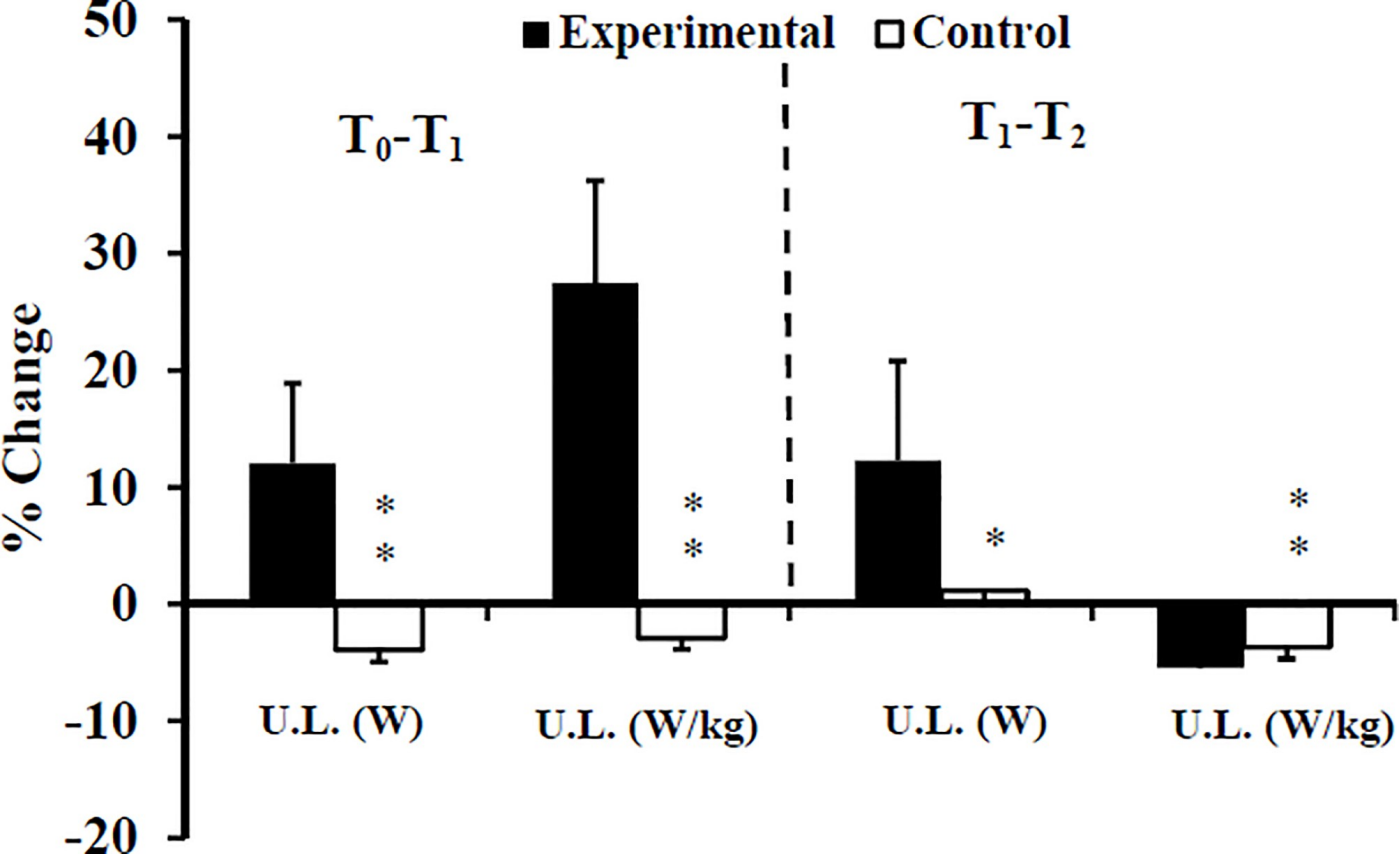
Percentage changes of power of upper limb at T_1_, and T_2_ for Experimental (E) and Control (C) groups. T_0_: before training; T_1_: after 10 weeks of resistance training; T_2_: after 2 weeks of tapering; U.L: upper limb; *: ANOVA group x time interaction significantly different between E and C at the level of *p* < 0.05; **: ANOVA group x time interaction significantly different between E and C at the level of *p* < 0.01.

**Fig 4:**
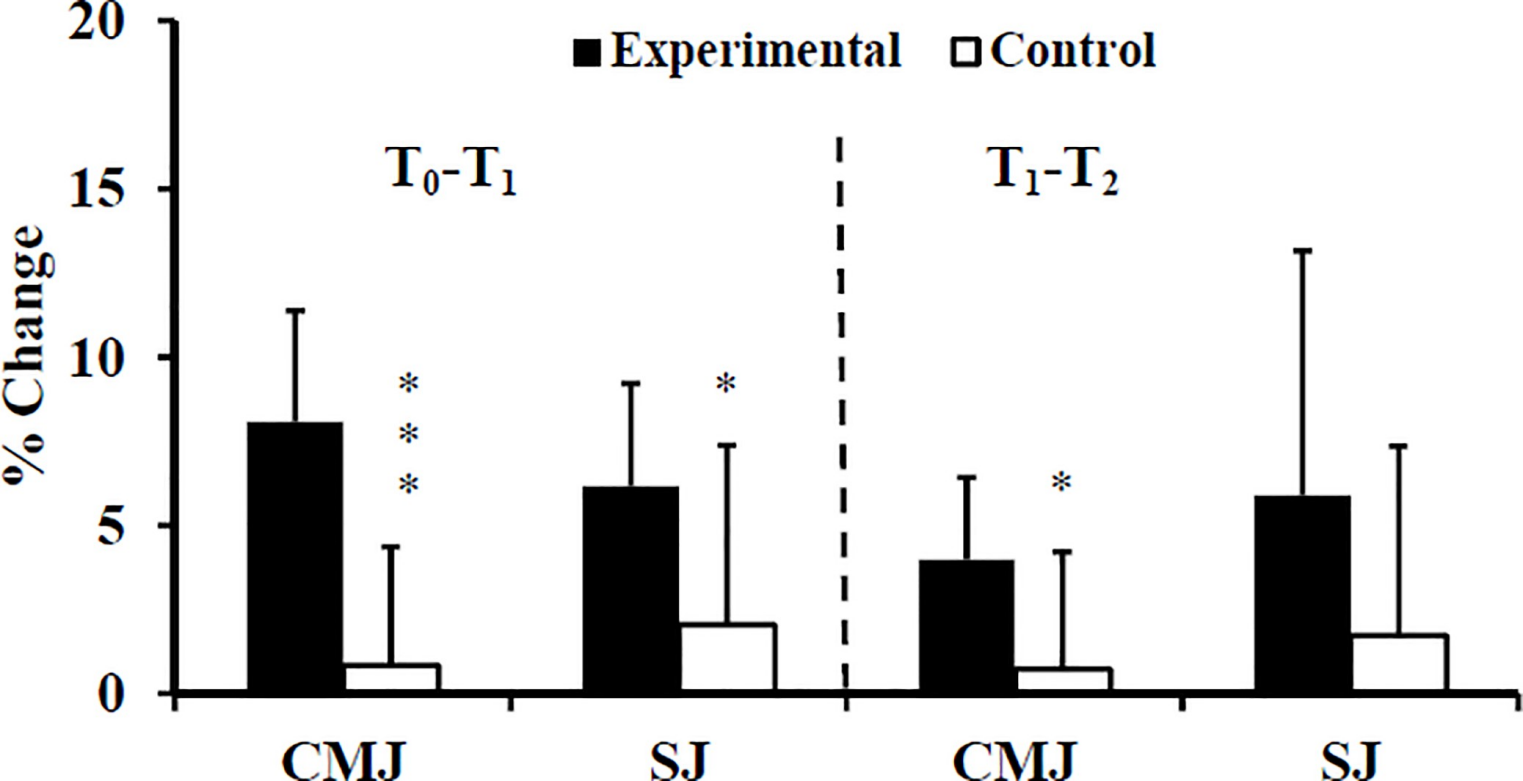
Percentage changes of vertical jump height at T_1_, and T_2_ for Experimental (E) and Control (C) groups. (C) groups. T_0_: before training; T_1_: after 10 weeks of resistance training; T_2_: after 2 weeks of tapering; CMJ: Counter-movement Jump; SJ: Squat Jump; *: ANOVA group x time interaction significantly different between E and C at the level of *p* < 0.05; ***: ANOVA group x time interaction significantly different between E and C at the level of *p* < 0.001.

**Fig 5:**
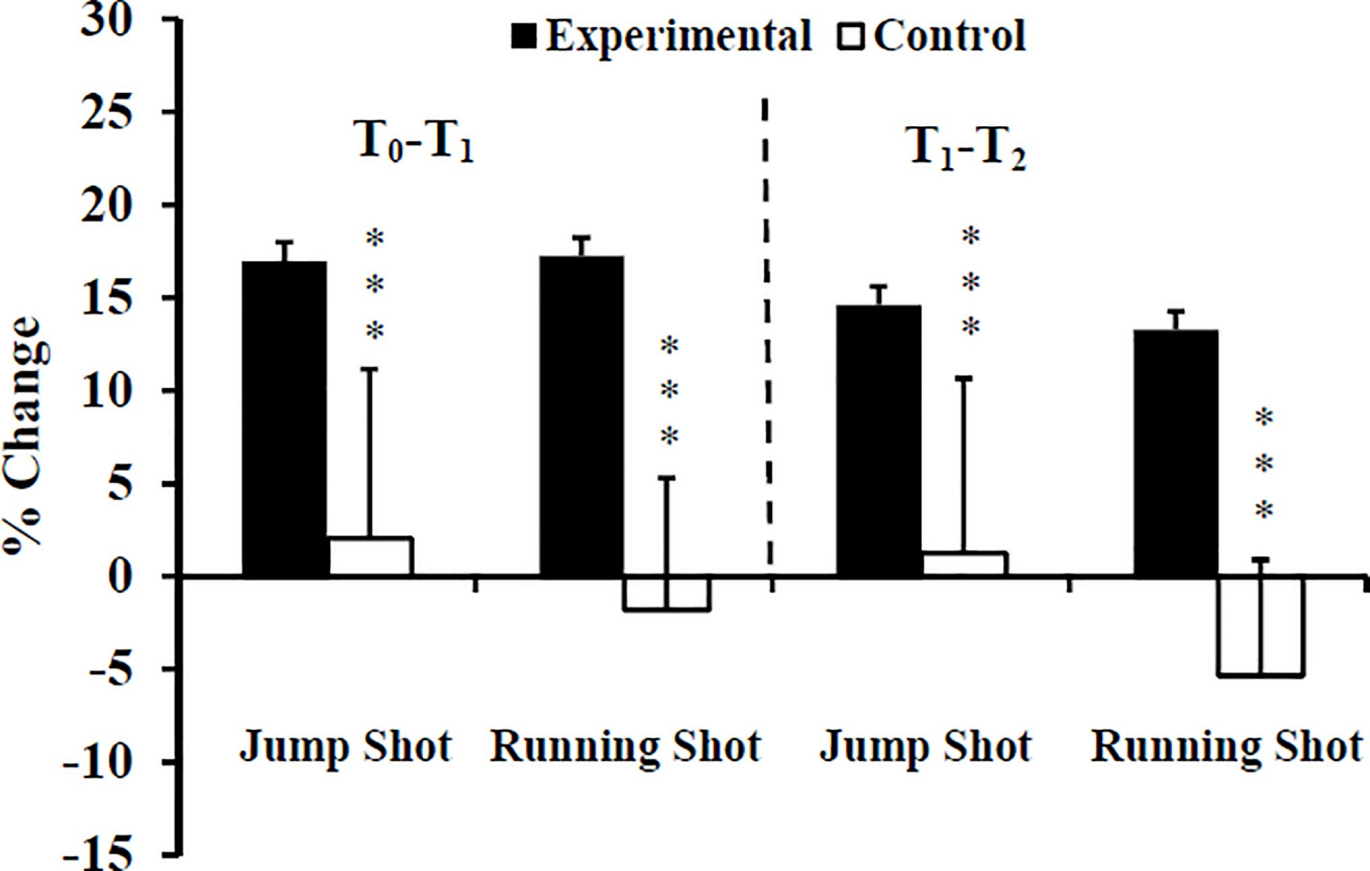
Percentage change of ball throwing velocity at T1, and T2 for Experimental (E) and Control (C) groups. T_0_: before training; T_1_: after 10 weeks of resistance training; T_2_: after 2 weeks of tapering; ***: ANOVA group x time interaction significantly different between E and C at the level of *p* < 0.001.
